# Single-Center Experience With Protocolized Treatment of Left Ventricular Assist Device Infections

**DOI:** 10.3389/fmed.2022.835765

**Published:** 2022-05-24

**Authors:** Nelianne J. Verkaik, Yunus C. Yalcin, Hannelore I. Bax, Alina A. Constantinescu, Jasper J. Brugts, Olivier C. Manintveld, Ozcan Birim, Peter D. Croughs, Ad J. J. C. Bogers, Kadir Caliskan

**Affiliations:** ^1^Department of Medical Microbiology and Infectious Diseases, Rotterdam, Netherlands; ^2^Department of Cardiology, Unit of Heart Failure, Heart Transplantation and Mechanical Circulatory Support, Rotterdam, Netherlands; ^3^Department of Cardio-Thoracic Surgery, Rotterdam, Netherlands; ^4^Department of Internal Medicine, Division of Infectious Diseases, Erasmus University Medical Center, Rotterdam, Netherlands

**Keywords:** left ventricular assist device (LVAD), antimicrobial treatment, *Staphylococcus aureus*, protocolized treatment, LVAD infections, heart-assist devices

## Abstract

**Purpose:**

Because of the current lack of evidence-based antimicrobial treatment guidelines, Left Ventricular Assist Device (LVAD) infections are often treated according to local insights. Here, we propose a flowchart for protocolized treatment, in order to improve outcome.

**Methods:**

The flowchart was composed based on literature, consensus and expert opinion statements. It includes choice, dosage and duration of antibiotics, and indications for suppressive therapy, with particular focus on *Staphylococcus aureus* (SA) (Figure 1). The preliminary treatment results of 28 patients (2 from start cephalexin suppressive therapy) after implementation in July 2018 are described.

**Results:**

Cumulative incidence for first episode of infection in a 3-year time period was 27% (26 of 96 patients with an LVAD). Twenty-one of 23 (91%) first episodes of driveline infection (10 superficial and 13 deep; nine of 13 caused by SA) were successfully treated with antibiotics according to flowchart with complete resolution of clinical signs and symptoms. For two patients with deep driveline infections, surgery was needed in addition. There were no relapses of deep driveline infections, and only 2 SA deep driveline re-infections after 6 months. Nine patients received cephalexin of whom four patients (44%) developed a breakthrough infection with cephalexin-resistant gram-negative bacteria.

**Conclusions:**

The first results of this protocolized treatment approach of LVAD infections are promising. Yet, initiation of cephalexin suppressive therapy should be carefully considered given the occurrence of infections with resistant micro-organisms. The long-term outcome of this approach needs to be established in a larger number of patients, preferably in a multi-center setting.

## Introduction

Continuous-flow left ventricular assist devices (LVADs) are a well-recognized treatment option for end-stage heart failure, with major benefits in quality of life and survival (1, 2). However, infections frequently occur with an incidence as high as 25–30% in the first 2 years (1, 3). LVAD-specific infections are serious with considerable rates of morbidity and mortality (3, 4).

In 2013, an expert opinion statement for the treatment of LVAD infections was published (5). In addition, a consensus statement from the International Society for Heart and Lung Transplantation (ISHLT) on the treatment of mechanical circulatory support infections was released in 2017 (6). Although these documents are a major step forward in the diagnosis and treatment of LVAD infections, the therapeutic options are not specified with respect to choice and dosages of antibiotics and could be more specified with respect to treatment duration. In addition, there is no guidance as to the treatment of recurrent infections. Consequently, LVAD infections are often treated according to local physicians' insights and experience with significant interclinical variability. As such, the lack of a standardized approach might contribute to the high recurrence rates reported (7). Here, we propose a flowchart for protocolized treatment of LVAD infections, based on available consensus and expert opinion statements, literature, and rationale. Our preliminary treatment results after implementation of the flowchart are described.

## Methods

A detailed flowchart for the treatment of LVAD infections was developed including suggestions for antibiotic drug of choice, dosage, and treatment duration. The recommendations in the flowchart were based on a combination of retrospective data and clinical experience from our center, available literature including the ISHLT consensus document and the Mayo Clinic expert opinion (5, 6) together with the Infectious Diseases Society of America Practice (IDSA) Guidelines for the Diagnosis and Management of Skin and Soft Tissue Infections (8). Superficial and deep driveline infections were defined according to Hannan et al. (9) Relapse or re-infection definitions were based on infective endocarditis literature (10), since these definitions are not established in the context of LVAD infections yet. The treatment of LVAD infections according to the flowchart started in July 2018 (with doxycycline as 2^nd^ empirical choice instead of co-trimoxazole from October 2019 onwards). Our preliminary treatment results after implementation of the flowchart are described till November 2021. Individual consent forms have been signed by all patients included and data collection was approved as a part of the European Registry for Patients with Mechanical Circulatory Support (EUROMACS) by the internal ethics committee.

### Definitions

- Superficial/exit site infection: minimal erythema spreading around the exit site
- Deep driveline infection: involves deep soft tissue (e.g., fascial and muscle layers)
- Relapse: a second superficial LVAD driveline infection or second deep LVAD driveline infection caused by the same species *within* 6 months after start of treatment of the initial episode
- Re-infection: a second superficial LVAD driveline infection or second deep LVAD driveline infection caused by the same species *after* 6 months after start of treatment of the initial episode
- Second episode of infection: general term for a new episode of infection
- Presumed *S. aureus* infection: pathogen not cultured, but patient clinically responding to antistaphylococcal therapy

## Results

### Rationale for the Proposed Flowchart for Protocolized Treatment of LVAD Infections

**Figure 1:**
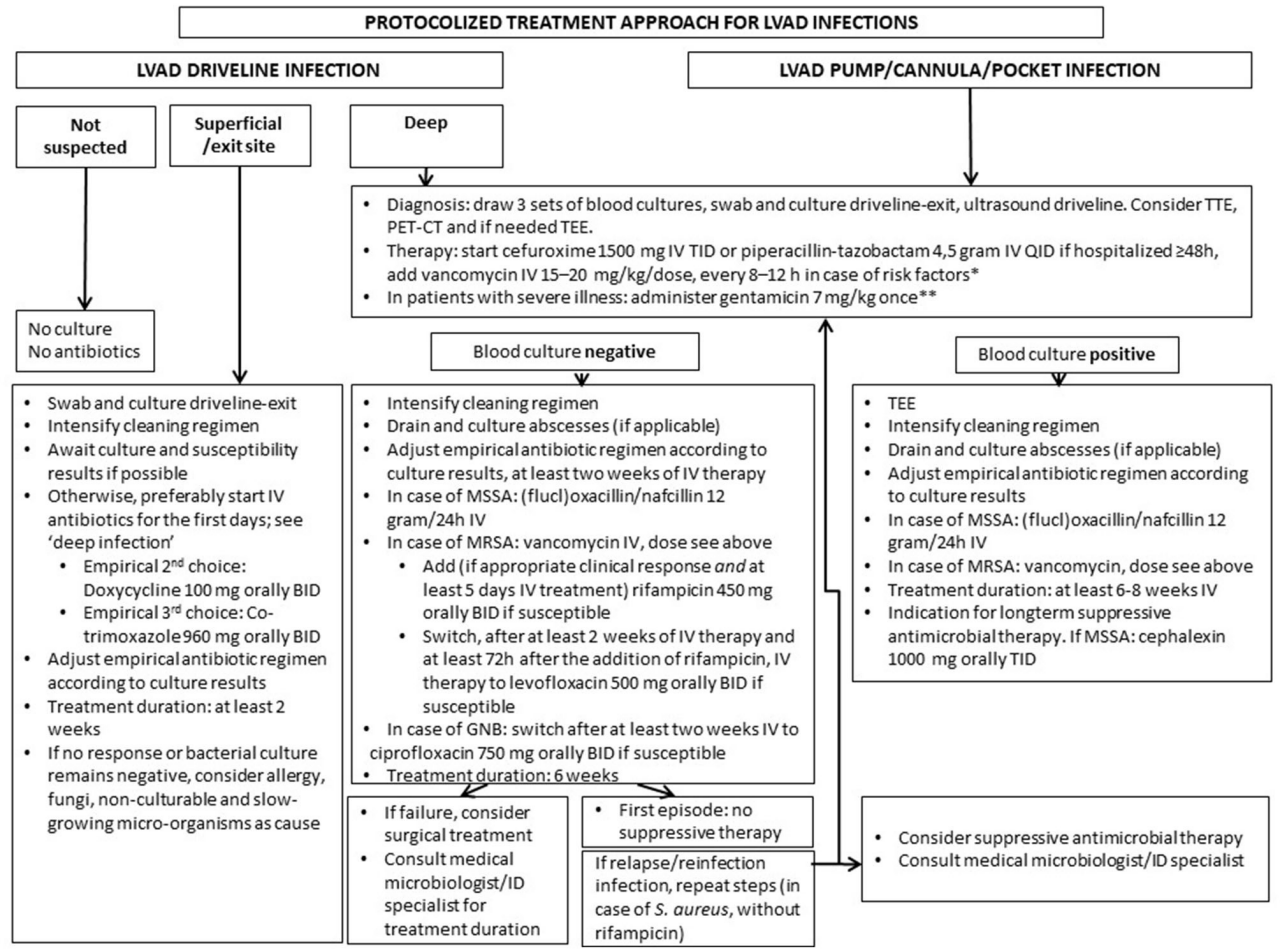
Proposed protocolized treatment approach for LVAD infections. Remarks: for deep driveline and LVAD pump/cannula/pocket infection surgical intervention may be required. Always take into account prior culture and susceptibility results. Dosages of antibiotics are based on a normal GFR. Check if dosage needs to be adjusted in case of decreased GFR or (morbid) obesity (in the Netherlands, according to the Dutch Working Party on Antibiotic Policy guidelines). *nasal colonization or prior positive cultures or infection with MRSA, countries where MRSA prevalence is high; **if GFR <30 ml/min, do not administer gentamicin, in patients with severe illness start meropenem iv 1,000 mg BID. BID, twice a day; TID, three times a day; QID, four times a day; TEE, trans-esophageal echocardiography; TTE, trans-thoracic echocardiography; PET-CT, positron emission tomography computed tomography; kg, kilogram; IV, intravenously; mg, milligram; GNB, gram negative bacteria; GFR, glomerular filtration rate; MSSA, methicillin susceptible *Staphylococcus aureus*; MRSA, methicillin-resistant *Staphylococcus aureus*.

Retrospective data on LVAD infections in our own center identified *S. aureus* as the most frequently isolated micro-organism in patients with driveline infections, in line with previous reports (5, 11, 12). In addition, the highest recurrence rate was found in patients with *S. aureus* driveline infection. This prompted us to develop a protocolized treatment approach with particular focus on *S. aureus* (Figure 1). Choices of drugs are based on the IDSA Practice Guidelines for the Diagnosis and Management of Skin and Soft Tissue Infections (8). Duration of therapy is based on the existing expert opinion and consensus documents (5, 6). We advocate starting with a high-dose, preferably intravenous, antimicrobial regimen in the first days in order to reach adequate drugs levels at the infection site. A number of issues need to be addressed specifically.

First, in the Netherlands, the prevalence of methicillin-resistant *S. aureus* (MRSA) carriage is low (at hospital admission, between 0.03 and 0.17%) (13) and so far in our center, only one MRSA LVAD driveline infection has been identified. However, in countries where MRSA prevalence is higher, antimicrobial agents with activity against MRSA should be part of the empirical regimen in case of severe LVAD infections as specified in the flowchart.

Second, the addition of rifampicin to an antistaphylococcal agent is advised because of its excellent antibiofilm activity, as suggested reasonable to be considered by Zinoviev (14, 15). This drug combination in our view is essential for eradication and clearance in the first episode of deep driveline infection. However, rifampicin use warrants strict monitoring of international normalized ratio (INR) given the interaction with vitamin K antagonists. The same holds true when choosing co-trimoxazole as an oral de-escalation strategy. In our center, most patients can monitor INR by themselves. If monitoring was not possible, rifampicin was either not advised or subcutaneous nadroparine was given instead of vitamin K antagonists. In case of recurrent infection, rifampicin administration is not advised because an additive benefit of rifampicin is expected particularly in the early stage of biofilm formation (16).

Third, the duration for deep driveline infections in the flowchart is set for 6 weeks, and 2 weeks for superficial driveline infections, based on the ISHLT consensus document (6). Nienaber et al. suggest 2–4 weeks of treatment, but no distinction is made between superficial and deep driveline infections (5).

Fourth, although suppressive antimicrobial treatment may be considered in the setting of deep driveline infections, there are conflicting data regarding its impact on relapse and superinfection with resistant micro-organisms, as stated in the ISHLT consensus (6). Therefore, suppressive therapy was recommended in the original protocol in case of recurrent infection only. In case of recurrent MSSA infection, cephalexin 1,000 mg TID was proposed based on a study showing successful suppression of MSSA LVAD infections, in contrast to clindamycin (17). However, the number of breakthrough infections with resistant gram-negative micro-organisms (details in preliminary results section) in patients on cephalexin suppressive therapy prompted us to adjust the flowchart to “consider suppressive antimicrobial therapy.” In case of pump infections, the ISHLT consensus supports the use of oral suppression therapy, which is recently suggested as a reasonable approach also, since patients presenting with LVAD-related blood stream infection are at high risk of relapse (18). Therefore, for pump infections, oral suppressive therapy after intravenous therapy is advised in the protocol.

### Preliminary Results After Implementation of the Protocolized Treatment Approach

Since July 2018, 28 patients with LVAD infections have been managed accordingly (Table 1). Two patients from start of suppressive therapy, twenty-six patients from start of treatment of first episode of infection (*n* = 23 driveline infections of which *n* = 13 deep infections, *n* = 3 pump infections). The cumulative incidence of the first episode of infection was 27% in 3-year time period (26 of 96 patients with an LVAD in follow-up).

**Table 1:** Preliminary results of LVAD patients who received protocolized treatment according to flowchart.

**Patients (*n*)**	**28**
Male (*n*)	25 (89%)
Female (*n*)	3 (11%)
Median body mass index (kg/m^2^) [range]	26 (18–38)
Diabetes mellitus (*n*)	7
**First episode of infection (** ***n*** **)**	**26**
Superficial driveline infection (*n*)	10 (38%)
Deep driveline infection (*n*)	13 (50%)
* (presumed) S. aureus (8 MSSA, 1 MRSA)	9
* Gram negatives (P. mirabilis, S. marcescens, K. pneumoniae, P. aeruginosa)	4
Pump infection (*n*)	3 (12%)
* S. aureus	2
* E. faecalis	1
Antibiotic therapy only (*n*)	24 (92%)
Median number of days [range] between LVAD implantation-first episode of infection	530 [57–1945]
Median age [range] at first episode (years)	60 [32–78]
**Second episode of infection (** ***n*** **)**	**8**
Superficial driveline infection (*n*)	4 (50%)
*of which relapse*	2
*of which re-infection*	2
Deep driveline infection (*n*)	4 (50%)
*(presumed) S. aureus	4
*of which relapse*	0
*of which re-infection*	2
Antibiotic therapy only (*n*)	7
Antibiotic therapy in combination with surgery (*n*)	1
Median number of days [range] between first and second episode	206 [94–761]
**Cephalexin suppressive therapy (** ***n*** **)**	**9**
No breaktrough infection (*n*)	4 (44%)
Follow-up duration (days) #	585 [450–1080]
Breaktrough infection (*n*)	4 (44%)
*A. baumanni complex	2
*P. mirabilis	1
*P. aeruginosa	1
Median number of days [range] from start cephalexin	150 [75–273]
Antibiotic therapy in combination with surgery for breakthrough infection (*n*)	3
Side effects leading to discontinuation of cephalexin (*n*)	1 (11%)
Number of days from start cephalexin	170

*#one patient was transplanted at day 450*.

Antimicrobial treatment of driveline infections resulted in resolution of signs and symptoms for 21/23 patients (91%). For two patients, surgical relocation was needed (one patient with *Pseudomonas aeruginosa*, one patient with *Serratia marcescens* deep driveline infection). Eight of nine patients with (presumed) *S. aureus* deep driveline infection were additionally treated with rifampicin of whom one patient stopped rifampicin due to rash after 18 days. There were no intractable complications caused by the interaction between rifampicin and cumarin derivatives.

A second episode of infection occurred in eight patients (*n* = 4 superficial, *n* = 4 deep) after a median of 206 days (range 94–761) days. Of the four superficial driveline infections, two were relapses and two were re-infections (pathogen not cultured). There were four (presumed) MSSA deep driveline infections, of which two were re-infection after 192 days and 351 days, respectively. There were no relapses of deep driveline infection. Three of 13 patients treated with levofloxacin for (presumed) *S. aureus* driveline infection, suffered from tendinitis/myalgia. After switching levofloxacin to another antimicrobial drug, the tendinitis-like symptoms resolved in all patients.

### Suppressive Therapy

Nine patients with *S. aureus* infection received cephalexin suppressive therapy 1,000 mg TID (*n* = 4 *S. aureus* deep driveline infection, *n* = 2 pump infections and *n* = 3 suppressive therapy as a clinical decision, off protocol). Although cephalexin was successful in suppression of *S. aureus* infection in all patients, including the two patients with *S. aureus* pump infection, four patients (44%) developed a breakthrough infection with a cephalexin resistant micro-organism and one patient switched from cephalexin to flucloxacillin because of nausea after 170 days. This patient also stopped flucloxacillin because of intolerance and still is infection-free after 600 days.

## Discussion

The current lack of evidence-based antimicrobial treatment guidelines hampers optimal treatment of LVAD infections. Therefore, a detailed flowchart to treat LVAD infections is proposed. In our center, it resulted in a more uniform antimicrobial treatment approach of LVAD infections. Antimicrobial treatment according to this protocolled to resolution of clinical signs and symptoms in the majority (91%) of patients. There were no relapses of deep driveline infections and only one presumed and one proven MSSA deep driveline re-infection were observed. The long duration between the first and second episode in these patients (192 days and 1 year, respectively) argue in our view against failure of the original antimicrobial regimen as a cause. These promising preliminary results seem to support the use of rifampicin in this context.

Suppressive therapy with cephalexin was not as successful as anticipated. Four (44%) patients, although successfully suppressed for *S. aureus*, developed a driveline infection with a cephalexin resistant micro-organism. While a causative relation with cephalexin use is hard to prove, this might have been a contributive factor. In literature, there are conflicting data regarding the clinical efficacy and risk of superinfection associated with suppressive antimicrobial therapy in patients with LVAD infections (6). Based on our data, we underscore that suppressive therapy, particularly in case of driveline infection, should be used restrictively, carefully weighing the chance of success against the risk of side effects and consequences of breakthrough infection with cephalexin resistant micro-organisms for which remaining treatment options might be limited. For patients with LVAD-related bloodstream infection, because of the high risk of relapse, suppressive therapy is considered reasonable (18).

The limitations of this study are its single-center and observational design. Only a limited number of patients are included. Although there is a considerable follow-up of more than 3 years from the implementation of the flowchart, outcome on the long-term is still uncertain. The majority of infections were driveline related, and we only have experience with one MRSA and only few gram-negative driveline infections. A protocol for additional local therapy would have been valuable. Since experience with antimicrobials and antimicrobial sensitivities may differ from one institution to the other, it may be challenging to institute the same protocol. Nevertheless, we believe this protocolized approach could serve as a framework for other LVAD treatment centers since *S. aureus* is the predominant causative micro-organism worldwide (5, 6, 11, 12).

In conclusion, the first results of the proposed protocolized treatment approach of LVAD infections are promising. Yet, initiation of suppressive therapy with cephalexin should be carefully considered given the occurrence of infections with resistant micro-organisms. A longer period of clinical experience and inclusion of more patients, preferably multi-center, are needed to establish the long-term outcome of this approach.

## Data Availability Statement

The datasets presented in this article are not readily available because the study is ongoing. Requests to access the datasets should be directed to k.caliskan@erasmusmc.nl.

## Ethics Statement

Data collection was approved as a part of the European Registry for Patients with Mechanical Circulatory Support (EUROMACS) by the internal Ethics Committee. The patients provided their written informed consent to participate in this study.

## Disclosure

The data was partly presented as a poster at the IDWeek 2019, Washington, United States of America.

## Author Contributions

NV and YY were part of the first draft and data analysis. NV and KC were part of the conceptualization of the study. NV, YY, HB, AC, JB, OM, OB, PC, AB, and KC were part of the data curation, patient care, and critical reviewers of the first draft. All authors contributed to the article and approved the submitted version.

## Conflict of Interest

The authors declare that the research was conducted in the absence of any commercial or financial relationships that could be construed as a potential conflict of interest.

## Publisher's Note

All claims expressed in this article are solely those of the authors and do not necessarily represent those of their affiliated organizations, or those of the publisher, the editors and the reviewers. Any product that may be evaluated in this article, or claim that may be made by its manufacturer, is not guaranteed or endorsed by the publisher.
